# Reflection on modern methods: constructing directed acyclic graphs (DAGs) with domain experts for health services research

**DOI:** 10.1093/ije/dyac135

**Published:** 2022-06-17

**Authors:** Daniela Rodrigues, Noemi Kreif, Anna Lawrence-Jones, Mauricio Barahona, Erik Mayer

**Affiliations:** NIHR Imperial Patient Safety Translational Research Centre, Institute of Global Health Innovation, Department of Surgery & Cancer, Imperial College London, London, UK; Centre for Health Economics, University of York, York, UK; NIHR Imperial Patient Safety Translational Research Centre, Institute of Global Health Innovation, Department of Surgery & Cancer, Imperial College London, London, UK; Centre for Mathematics of Precision Healthcare, Department of Mathematics, Imperial College London, London, UK; NIHR Imperial Patient Safety Translational Research Centre, Institute of Global Health Innovation, Department of Surgery & Cancer, Imperial College London, London, UK

**Keywords:** Causal inference, potential outcomes, directed acyclic graphs, policy evaluation, health services research

## Abstract

Directed acyclic graphs (DAGs) are a useful tool to represent, in a graphical format, researchers’ assumptions about the causal structure among variables while providing a rationale for the choice of confounding variables to adjust for. With origins in the field of probabilistic graphical modelling, DAGs are yet to be widely adopted in applied health research, where causal assumptions are frequently made for the purpose of evaluating health services initiatives. In this context, there is still limited practical guidance on how to construct and use DAGs. Some progress has recently been made in terms of building DAGs based on studies from the literature, but an area that has received less attention is how to create DAGs from information provided by domain experts, an approach of particular importance when there is limited published information about the intervention under study. This approach offers the opportunity for findings to be more robust and relevant to patients, carers and the public, and more likely to inform policy and clinical practice. This article draws lessons from a stakeholder workshop involving patients, health care professionals, researchers, commissioners and representatives from industry, whose objective was to draw DAGs for a complex intervention—online consultation, i.e. written exchange between the patient and health care professional using an online system—in the context of the English National Health Service. We provide some initial, practical guidance to those interested in engaging with domain experts to develop DAGs.

Key Messages

- Directed acyclic graphs (DAGs) can be used as a graphical tool to represent researchers’ assumptions about the causal structure among variables while providing a rationale for the choice of confounding variables to adjust for.
- Despite their rise in popularity, the lack of practical guidance on how to create and use DAGs in applied health research has limited their wide adoption in the field.
- Some progress has recently been made in terms of building DAGs based on studies from the literature, but there remains the need for concrete examples on how to construct DAGs with domain experts.
- Building DAGs with domain experts, as opposed to having DAGs solely based on researchers’ understanding of the literature, can promote a more robust and realistic representation of the causal model under study.

At the core of a ‘learning health system’ is the capacity to assess ‘what works’, or more formally, to estimate the causal effect of interventions on their expected outcomes. Early qualitative frameworks for causal inference in epidemiology include the Bradford Hill criteria,^1^ the sufficient-component cause model,^2^^,^^3^ inference to the best explanation^4^ and triangulation.^5^^,^^6^ In recent decades, however, there has been a move towards a quantitative approach to causal inference^7^ through the use of the potential outcomes framework, also known as the Neyman–Rubin model.^8–10^ The potential outcomes framework starts with the notion that each unit in the population can be characterized by a set of potential outcomes, one for each intervention level. In the real world at any point in time, only one of these potential outcomes is observed for each unit (also known as the ‘fundamental problem of causal inference’^11^). In general, causal effects at the individual level cannot be identified because of missing data on the counterfactual.^9^ When the focus is on population-level causal effects, there is a set of assumptions under which the causal estimand can be identified from the observed data. These include: no interference (the potential outcomes for any unit are not affected by other units’ treatment levels), consistency (the observed outcome is equal to the potential outcome at any treatment level), exchangeability (the potential outcomes are independent of treatment for any treatment level) and positivity (subgroups with similar characteristics have a non-zero probability of being in any intervention level).^12^^,^^13^

Double-blinded, randomized controlled trials (RCTs) with perfect compliance and no loss to follow-up are particularly effective in supporting the assumption of exchangeability, due to random assignment of treatment. However, most health services research is based on observational studies, in which the assignment mechanism that determines which units are assigned to which treatment levels is often unknown to the researchers, making it difficult to assume exchangeability. Under some degree of uncertainty it might be possible to assume conditional exchangeability, in other words that treatment was randomly assigned conditional on a set of variables.^12^ The variables to condition on have historically been selected based on statistical associations and/or by checking if each variable, individually, meets certain criteria for confounding (e.g. variable associated with the treatment and outcome, and not on the causal pathway between the treatment and outcome).^14^ More recently, researchers^14^^,^^15^ were able to illustrate how these historical confounder selection strategies are prone to bias, by showing that they may lead to important confounding variables to be missed from and/or non-confounding variables to be inadvertently included in the adjustment set. Consequently, they have argued that the choice of variables to condition on should be driven by the knowledge of the causal model under study. Directed acyclic graphs (DAGs), developed as part of the Pearl’s structural causal model framework,^16^^,^^17^ are a useful tool to represent, in a graphical format, researchers’ assumptions about the causal relationships between variables in a causal model^12^^,^^18^ (see Box 1). Additionally, DAGs coupled with the so-called backdoor criterion^16^^,^^17^ can provide a rationale for the choice of confounding variables to adjust for, increasing the credibility of the conditional exchangeability assumption. The backdoor criterion states that a set of variables is sufficient to control for confounding if it blocks all non-causal paths from treatment to the outcome and does not include any descendants of treatment such as mediators or colliders^16^^,^^17^ (see Box 2). This criterion has been fully automated in open-source software such as DAGitty [http://www.dagitty.net/] or Fusion [https://causalfusion.net/], which means it can easily be checked against any DAG, regardless of its complexity.

Box 1 Terminology in directed acyclic graphs (DAGs)

- A DAG is a collection of nodes (the dots) representing variables and directed edges (the arrows) connecting the nodes. Example:
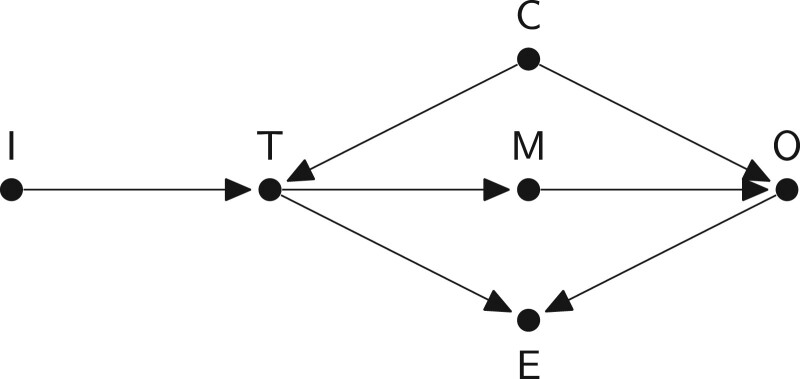
- In a causal DAG, the arrows represent causal relationships between variables, without specifying their sign, magnitude or form. In the example above, for example, variable C has an effect on both variable T and variable O.
- There are no directed cycles in a causal DAG because no variable can have a causal effect on itself at the same moment in time, and the future does not cause the past.

## Creating DAGs with domain experts

Despite their rise in popularity in recent years, DAGs are still ‘relatively rare’ in applied health research, as reported by Tennant *et al.*^19^^,^^20^ in recent reviews. The authors^19^^,^^20^ highlighted the limited practical guidance available on the development and use of DAGs, and the subsequent need for best practice to be developed. Recently, Ferguson *et al.*^21^ created a systematic approach to building DAGs based on studies from the literature. However, there remains the need for practical guidance on how to draw DAGs, based on the experience and knowledge of domain experts. This step is particularly relevant when the intervention under study is relatively new and for which there is minimal evidence from the literature. Additionally, incorporating both the perspectives of researchers (driven by the literature) and stakeholders of health services into DAGs, as opposed to having DAGs solely based on researchers’ understanding of the literature, can also make findings more robust and relevant to patients, carers and the public, and more likely to inform policy and clinical practice. With that in mind, we organized a workshop on drawing DAGs with domain experts for a complex intervention in the context of English primary care. We considered the simple setting of a one-time-point intervention, although the models could be extended to the longitudinal setting. Whereas we discussed mediators and instrumental variables, the main goal of the session was to identify the minimal adjustment set of variables that would be required to assume conditional exchangeability. For that purpose, we used the backdoor criterion. Finally, it is worth highlighting that DAGs could also be used to discuss other assumptions needed for causal inference, such as positivity and consistency^22^ as well as selection bias,^23^ measurement bias^24^ or time-dependent confounding,^25^ but these go beyond the scope of this work.

## The structure of the workshop

The online workshop ran for 2.5 h and included 20 domain-expert participants and three facilitators. The intervention under study was online consultation in English primary care, i.e. written exchange between the patient and health care professional using an online system,^26^ which contrasts with the more traditional, in-person or telephone consultation. The domain experts included three patients, six health care professionals (including two general practitioners, a nurse, a pharmacist, a practice manager and an administrator), six researchers, three commissioners from the NHS and two representatives from industry. This group was chosen because of their knowledge, experience and/or interest in online consulting in English primary care. Prior to the workshop, a document with information about the research project and DAGs was shared with all participants. On the day of the workshop, after a short presentation about the project and DAG methodology, the group was split into three breakout rooms, each room with mixed expertise and a facilitator. To make sure all breakout sessions would follow a similar structure, a set of questions to guide the discussions was prepared beforehand. There were two 30-min breakout sessions: the first dedicated to the ‘brainstorming’ phase and the second to the ‘refinement’ phase. After each breakout session, the group got back together for 15 min to share and discuss their proposed DAGs. We used Microsoft PowerPoint to create the DAGs during the workshop, but our recommendation would be to use DAGitty or Fusion software instead, as explained below.

## Key information for domain experts to consider during DAG development

Domain experts do not need to fully understand the mathematical graph theory underpinning DAGs, but it might be important to highlight the rationale of their use in health services research. In addition, participants involved in DAG development might find it helpful to understand their basic terminology (Box 1), the key types of variables involved (Box 2) and more importantly, some of the key considerations in their development (Box 3). Participants might also find it useful to see examples of DAGs published in peer-reviewed journals via [causaldiagrams.org], a database that allows for filtering studies by exposure and outcome, among others. [DAGBase.net] is another available database where it is possible to search for DAGs created using DAGitty software.

Box 2 Key types of variables that can be found in a causal directed acyclic graph (DAG)

1. Treatment/intervention/exposure (T): the main cause.
2. Outcome (O): the main effect.
3. Mediator (M): caused by the treatment which in turn causes the outcome.
4. Confounder (C): common cause of the treatment and outcome.
5. Collider (E): common effect of any two variables on a backdoor path.*
6. Instrument (I): only causes the treatment (and not the outcome).

*Non-causal path from the treatment to the outcome.

Box 3 Key considerations when drawing a causal directed acyclic graph (DAG)

1. Variables should be drawn independently of available data.
2. Variables should be specific (e.g. ‘years of schooling’ instead of ‘education’) and measurable. This is particularly important for the treatment and outcome variables, but less so for mediators in case these are included only to aid understanding of potential treatment mechanisms.
3. Variables should be time-ordered (left-to-right or top-to-bottom), although other arrangements might be preferable in some cases.
4. Assumptions are encoded by the absence of an arrow between any two variables. A priori, all variables are interconnected. In the context of a DAG with missing arrows, it is important to be explicit that those arrows were intentionally removed and not something that was forgotten or not discussed.
5. The total effect of an intervention on a particular outcome—which includes both direct and indirect effects through mediators—is often the parameter of interest. In that context, there is no need to specify any mediators of the outcome in the DAG. However, when the intervention and proposed outcome do not have an obvious direct connection, it can be useful to specify at least one mechanism through which the intervention can lead to the outcome.

## The process of DAG development with domain experts

The process of DAG development with domain experts (Box 4) spanned four phases: brainstorming, refinement, exposition and reconciliation. The goal of brainstorming was to create the first draft of the DAG with multiple expected outcomes, followed by the refinement of that initial DAG by focusing on a specific outcome. In exposition, the aim was to obtain feedback from participants on the literature-driven DAGs created by the research team, whereas in reconciliation, features of all DAGs proposed by participants and the research team were combined into a final set of DAGs to be considered in the research project. After that, we applied the backdoor criterion to the final set of DAGs to find the minimal adjustment set of variables that would be required to obtain unconfounded effect estimates in this simple setting of a one-time-point intervention. It is worth noting, however, that in practice it might not be possible to remove confounding completely in observational studies because some key confounding variables might be unknown, and thus missing from the adjustment set and/or not measured in existing databases.^27^

**Box 4:** Framework for directed acyclic graph (DAG) development with domain experts

Phase	Aim	Actions	Questions
Brainstorming	To create the first draft of the DAG with multiple expected outcomes	Ensure there is a clear definition of the intervention under study and confirm that participants understand it Include all expected outcomes of the intervention. If the research team has only access to routinely collected data and primary data collection is out of consideration, ask participants to focus on outcomes that are more likely to be captured in routine datasets. Start connecting intervention and outcome variables using arrows, and if possible include a written justification for each path on a side note Include all relevant factors that influence how the intervention is assigned. Start connecting those variables to the intervention variable using arrows, and if possible include a written justification for each path on a side note	Is it clear for everyone what is the intervention under study? Can someone explain it to the group? What relevant outcome variables do we need to add to the graph? What exactly are we expecting to achieve with this intervention? What are the key factors driving the assignment of the intervention?
Refinement	To refine the initial draft of the DAG by focusing on a specific outcome	Discuss the outcome with participants Starting from a saturated graph, ask participants if there are any arrows that can be omitted by assuming those variables are not causally related. Follow the temporal order depicted in the graph (e.g. from left to right) Confirm with participants that there are no other common causes of the intervention and outcome that need to be added to the graph If new common causes of the intervention and outcome are added to the graph, repeat step 2 for each new variable	Do the intervention and proposed outcome have an obvious direct connection? If not, could you think of a particular mechanism through which the intervention can lead to the proposed outcome? Which key mediators should be included? If we focus on <variable 1 name> for now, are there any arrows going from this variable that can be omitted? How confident are you that <variable 1 name> and <variable 2 name> are not causally related? Could anyone think of other common causes of the intervention and outcome that might be missing from the graph?
Exposition	To obtain feedback from participants on the DAGs created by the research team based on empirical evidence and theories from the literature	Explain the DAGs to participants Ask participants for feedback on the DAGs	2. Do you agree with the mechanism through which the intervention causes the outcome? Are there any arrows that we need to add/can remove? Could anyone think of other common causes of the intervention and outcome or factors that only cause the intervention to include in the graph?
Reconciliation	To analyse and, whenever appropriate, combine features of all DAGs proposed by participants and research team into a final set of DAGs to be considered in the research project	Include all confounding variables identified by both groups in the final set of DAGs Share all DAGs with participants for their final revision and validation and incorporate any feedback	

## Brainstorming

**Figure 1:**
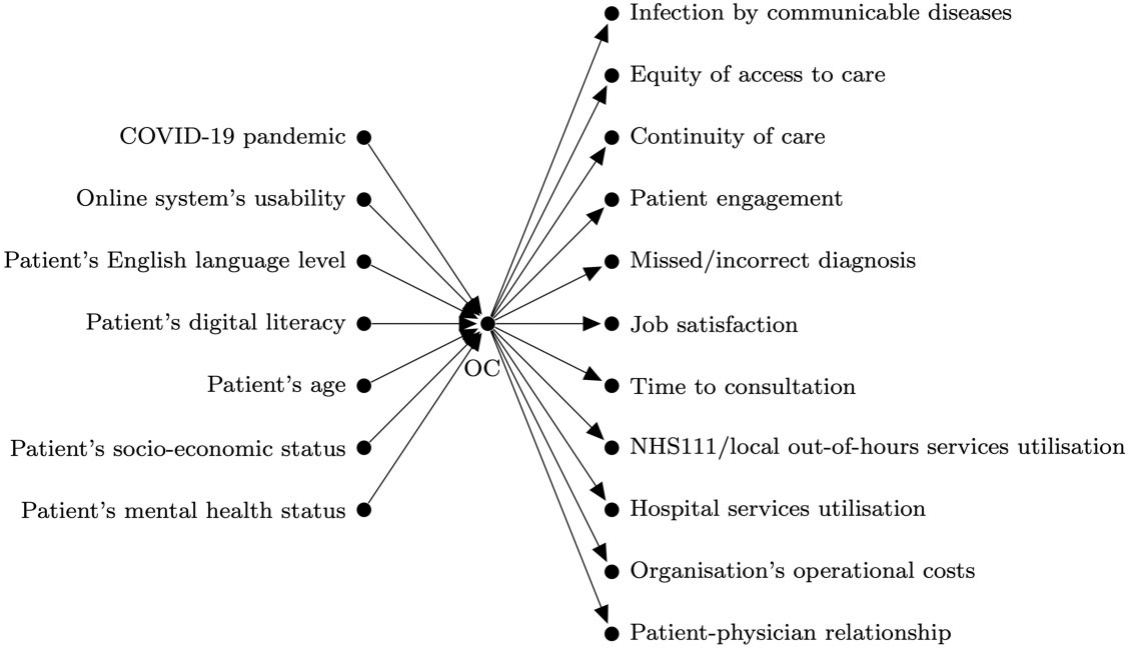
The first draft of the directed acyclic graph proposed by group A at the brainstorming phase (unsaturated version). The variable OC, which stands for online consultation, is the intervention under study.

In the brainstorming phase, each group created their first draft of the DAG with multiple expected outcomes. Figure 1 shows the unsaturated version of the DAG proposed by one group (group A). At this stage, we focused the discussion on expected outcomes, allocating less time to factors influencing the intervention. No time was dedicated to discussing the causal relationships between factors influencing the intervention or the relationships among outcomes.

## Refinement

**Figure 2:**
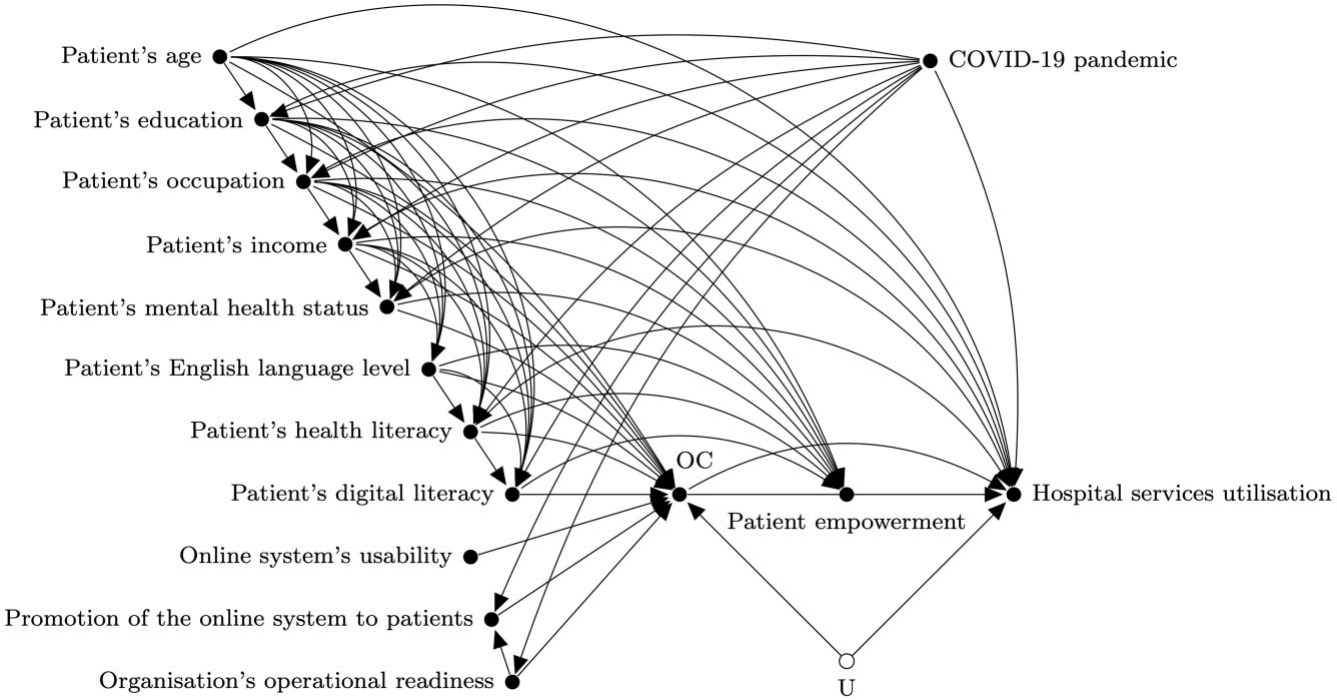
The complete directed acyclic graph proposed by group A at the refinement phase. The variable OC, which stands for online consultation, is the intervention and ‘hospital services utilisation’ is the outcome. The variable U illustrates unmeasured confounding which could not be ruled out due to time constraints.

The refinement phase aimed at improving the initial draft of the DAG by focusing on a specific outcome. Each group focused on a different outcome at this stage. Figure 2 shows the complete DAG proposed by group A, with ‘hospital services utilisation' as the outcome of interest. At this stage, we planned to start from a saturated graph and go through each variable while asking participants if there were any arrows that could be omitted. In practice, we ended up focusing the discussion on identifying confounders of the relationship between the intervention and outcome (e.g. patient’s age), but instruments (e.g. online system’s usability) and mediators (e.g. patient empowerment) also came up in the discussion. As a result, some arrows between those variables were missing at the end of the session, and consequently this step had to be completed by the research team. We then shared via e-mail the complete DAGs with participants for their final revision and validation.

## Exposition

**Figure 3:**
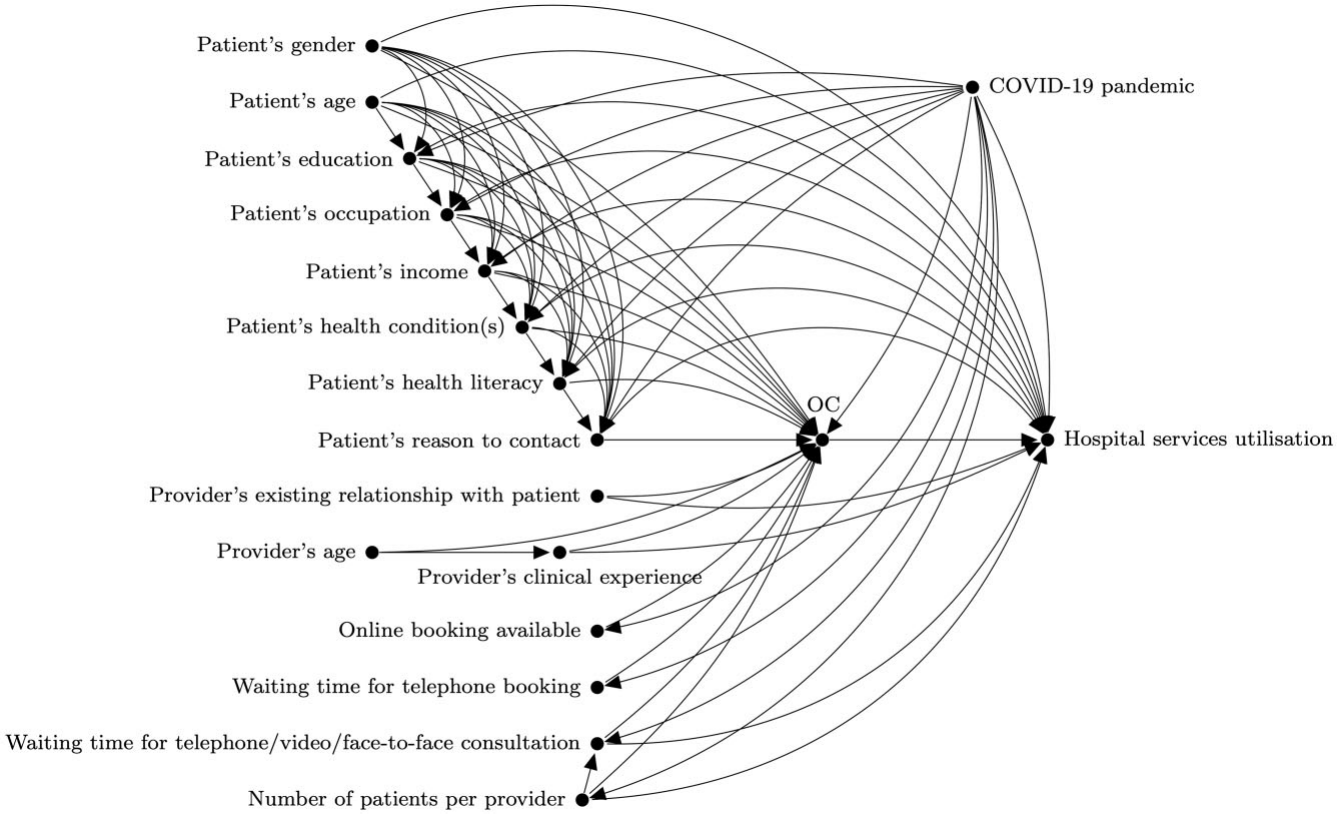
The directed acyclic graph created by the research team based on findings from the literature for the research question discussed by group A. The variable OC, which stands for online consultation, is the intervention and ‘hospital services utilisation’ is the outcome.

The purpose of the exposition phase was to generate debate around the DAGs created by the research team based on empirical evidence and theories from the literature. This was a two-step process. First, we searched for peer-reviewed articles on five academic databases (Embase, GlobalHealth, HMIC, Medline, PsycINFO) and grey literature. The search strategy included all patient groups, online consulting and related digital health interventions, and all domains of care quality. We skimmed through the abstracts and read relevant papers in full. The second step was guided by the method of Ferguson *et al.,*^21^ with potential relationships of interest from each relevant study being identified and DAGs drawn accordingly. Figure 3 shows the DAG created by the research team for the research question discussed by group A. Due to time constraints, this step was not covered during the workshop and the literature-driven DAGs were only shared via e-mail with participants after the event.

## Reconciliation

**Figure 4:**
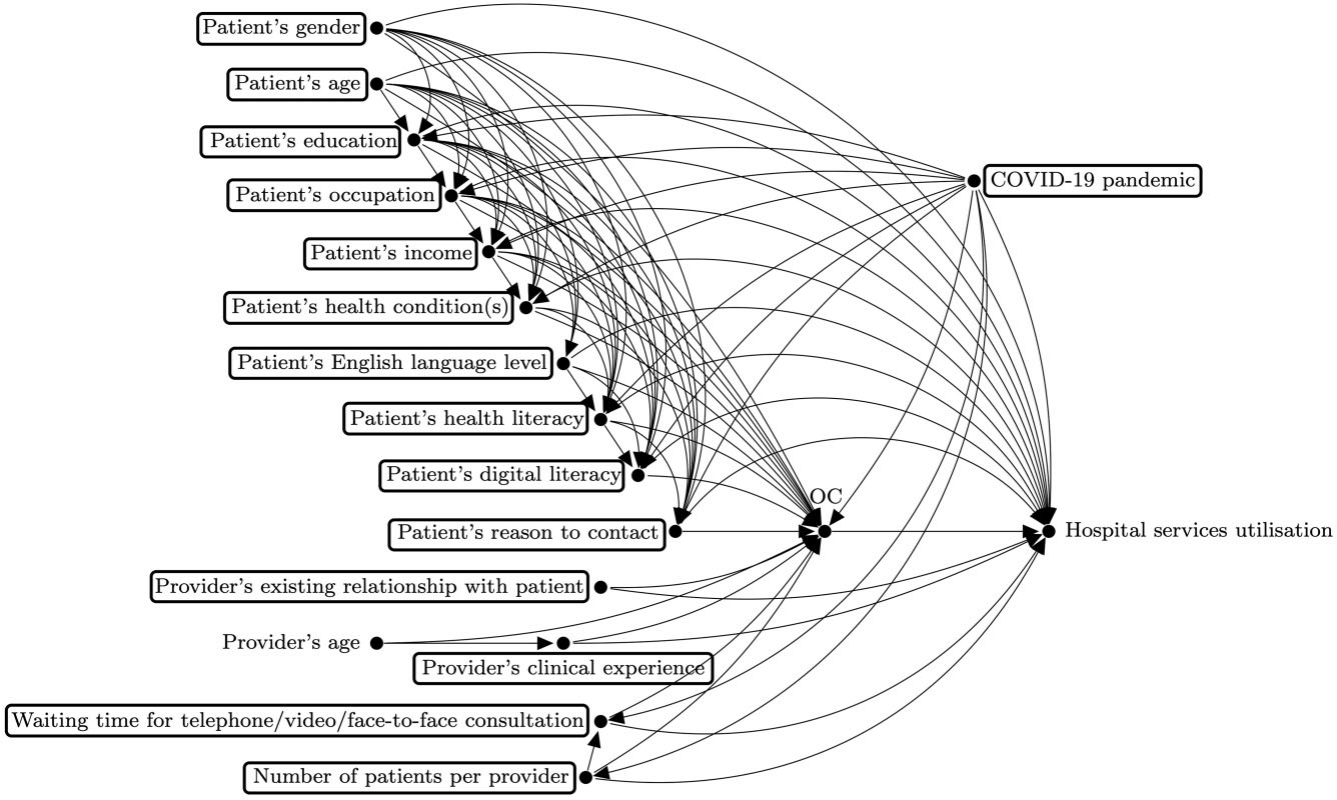
The final directed acyclic graph for the research question discussed by group A. The variable OC, which stands for online consultation, is the intervention and ‘hospital services utilisation’ is the outcome. Boxed variables constitute the minimal adjustment set according to the backdoor criterion.

The final phase, reconciliation, was completed by the research team after the workshop. Figure 4 shows the final DAG for the research question discussed by group A, with boxed variables constituting the minimal adjustment set based on the backdoor criterion. At this stage, we analysed and combined whenever appropriate the features of all DAGs from previous stages. We started by identifying seven confounding variables included by both groups: ‘patient’s age’, ‘patient’s education’, ‘patient’s occupation’, ‘patient’s income’, ‘patient’s health condition’, ‘patient’s health literacy’ and ‘COVID-19 pandemic’. This step was followed by the identification of eight additional confounding variables: ‘patient’s gender’, ‘patient’s reason to contact’, ‘provider’s existing relationship with patient’, ‘provider’s clinical experience’, ‘waiting time for telephone/video/face-to-face consultation’ and ‘number of patients per provider’ were included by the research team, but not domain experts; the opposite occurred for ‘patient’s English language level’ and ‘patient’s digital literacy’. In the final DAG, all 15 confounding variables were included, constituting the minimal adjustment set according to the backdoor criterion. Finally, we excluded instruments and mediators that were unlikely to be captured in routine datasets. The list of excluded instruments included ‘online system’s usability’, ‘promotion of the online system to patients’, ‘organization’s operational readiness’, ‘online booking available’ and ‘waiting time for telephone booking’. ‘Patient empowerment’ was the only mediator to be excluded. All DAGs were then shared via e-mail with participants for their final revision and validation, allowing 2 weeks for that.

## Lessons learned

In general the breakout sessions went according to plan, but there were some aspects that required adaptation. We chose Microsoft PowerPoint to create the DAGs because we envisioned an interactive workshop where all participants would interact with the DAG and draw their own arrows and variables during the workshop. This did not happen because it can be challenging for those with no prior knowledge of DAGs to create them on their own. Instead, it was up to the facilitator to draw the DAG while participants expressed their views. If feasible, future sessions could be facilitated by two people experienced in DAGs so that one could facilitate the discussion and the other could draw the DAG. In addition, it can be very difficult, in practice, to draw a complete DAG, with all confounding variables, mediators and instruments in one session. In the refinement phase, it could be beneficial to focus the discussion on confounding variables and the relationship between them. Moreover in preparation for the refinement phase, it can be useful if each facilitator connects all variables in each DAG without creating directed cycles, so that it is possible to start the discussion from a saturated graph. This can be more easily done in DAGitty or Fusion software, rather than Microsoft PowerPoint.

Finally, it is worth mentioning that the same research team created the literature-driven DAGs, synthesized the workshop discussions into the expert-driven DAGs and combined all features from both sources into the final set of DAGs. In an attempt to balance the contributions from the research team and domain experts, we planned to discuss the literature-driven DAGs with participants during the workshop, but due to time constraints, that was not possible. Instead, we shared with participants via e-mail all DAGs created at each stage of the framework and asked if the DAGs from the brainstorming and refinement phases were representative of the discussions held during the workshop, while also soliciting their feedback on the DAGs from the remaining phases. However, this resulted in few responses. If time permits, it could be valuable to go through the different phases of the framework for DAG development with domain experts over a number of sessions in order to enhance the robustness and relevance of the causal model under study.

## Final remarks

The rich discussions with stakeholders during the event allowed us, the research team, to become more familiar with the context and to understand in more detail the intervention under study, ultimately allowing us to make assumptions more in line with current practice. Whereas the literature-driven DAGs and expert-driven DAGs had several confounding variables in common, each set of DAGs also contributed with their own unique list of confounding variables. This highlights the benefits of involving stakeholders in health services research by promoting a more robust and realistic representation of the causal model under study.

In addition, it became clear that drawing DAGs and applying the backdoor criterion offer a practical, yet scientific approach to selecting the confounding variables to adjust for, increasing the credibility of the assumption of conditional exchangeability. Future sessions could focus on discussing other necessary assumptions for identifying causal effects^22^ and/or specific topics such as selection bias,^23^ measurement bias^24^ or time-dependent confounding.^25^

It goes without saying that creating DAGs with domain experts can be challenging. For example, it can be difficult to break down complex concepts into specific variables that are measurable; some of those variables might not be captured in existing datasets; and consensus is not always possible, which can result in a set of DAGs, instead of a unique DAG, to represent a specific relationship of interest. Nevertheless, we believe that these challenges can be informative by encouraging researchers to include proxies for certain variables while accounting for bias resulting from measurement error, and to conduct sensitivity analysis to check the robustness of results to different model specifications. In circumstances where it is not possible to measure all variables that are part of the minimal adjustment set, researchers might choose to perform sensitivity analysis for unmeasured confounding (e.g. using the E-value^28^^,^^29^), focus on partial identification of the estimand of interest through bounds^30^ or consider other identification strategies altogether (e.g. instrumental variable approach^13^). Finally, these challenges only highlight how important it is for researchers to be explicit about their causal model in order to promote research that is objective, transparent and reproducible.

## Ethics approval

Not applicable as no patient data were used.

## Data Availability

Not applicable as no specific data were used.
